# Biominerals and Bioinspired Materials in Biosensing: Recent Advancements and Applications

**DOI:** 10.3390/ijms25094678

**Published:** 2024-04-25

**Authors:** Mohamed A. A. Abdelhamid, Mi-Ran Ki, Seung Pil Pack

**Affiliations:** 1 Department of Biotechnology and Bioinformatics, Korea University, Sejong-ro 2511, Sejong 30019, Republic of Korea; mohamed42@korea.ac.kr (M.A.A.A.); allheart@korea.ac.kr (M.-R.K.); 2 Department of Botany and Microbiology, Faculty of Science, Minia University, Minia 61519, Egypt; 3 Institute of Industrial Technology, Korea University, Sejong-ro 2511, Sejong 30019, Republic of Korea

**Keywords:** biominerals, biomimetic materials, biosensor, sensitivity, selectivity, optical sensors, electrochemical sensors, magnetic biosensor, point-of-care diagnostics, environmental monitoring

## Abstract

Inspired by nature’s remarkable ability to form intricate minerals, researchers have unlocked transformative strategies for creating next-generation biosensors with exceptional sensitivity, selectivity, and biocompatibility. By mimicking how organisms orchestrate mineral growth, biomimetic and bioinspired materials are significantly impacting biosensor design. Engineered bioinspired materials offer distinct advantages over their natural counterparts, boasting superior tunability, precise controllability, and the ability to integrate specific functionalities for enhanced sensing capabilities. This remarkable versatility enables the construction of various biosensing platforms, including optical sensors, electrochemical sensors, magnetic biosensors, and nucleic acid detection platforms, for diverse applications. Additionally, bioinspired materials facilitate the development of smartphone-assisted biosensing platforms, offering user-friendly and portable diagnostic tools for point-of-care applications. This review comprehensively explores the utilization of naturally occurring and engineered biominerals and materials for diverse biosensing applications. We highlight the fabrication and design strategies that tailor their functionalities to address specific biosensing needs. This in-depth exploration underscores the transformative potential of biominerals and materials in revolutionizing biosensing, paving the way for advancements in healthcare, environmental monitoring, and other critical fields.

## 1. Introduction

The relentless pursuit of biosensing platforms with exceptional sensitivity, selectivity, and adaptability continues to drive advancements in various fields [1]. From diagnostics and environmental monitoring to cutting-edge biomedical research, these platforms play a crucial role in early disease detection, pollutant analysis, and personalized treatment strategies [2]. In this context, biomineral-based biosensors have emerged as a thriving area of exploration at the intersection of biomimicry and materials science. This rapidly evolving field draws inspiration from nature’s remarkable ability to orchestrate the formation of complex and exquisitely structured biominerals [3]. Examples include silica biominerals found in diatoms and sponges [4], calcium carbonate shells of mollusks and coccolithophores [5,6], magnetosomes within magnetotactic bacteria [7], and hydroxyapatite in bones and teeth [8]. These biominerals possess unique physicochemical properties that offer significant potential for biosensing applications.

By harnessing the principles of biomineralization (or biomimetic processes), researchers can engineer biosensors with unparalleled capabilities. This approach often involves mimicking, but not necessarily replicating, nature’s intricate processes to create bioinspired mineral structures [9,10]. These structures offer a high degree of control over material properties, enabling the design of biosensors that significantly surpass traditional methods in sensitivity, selectivity, and overall functionality.

The inherent properties of biominerals are strategically exploited to design innovative biosensors for diverse sensing applications. Recent breakthroughs showcase the versatility of this approach across various transduction mechanisms. Surface-enhanced Raman spectroscopy (SERS) benefits from biomineralization principles with the creation of biocompatible and highly sensitive SERS substrates [11,12]. In the realm of immunosensors, biominerals facilitate the development of highly specific and selective platforms for disease marker detection [13]. Bioinspired mineral structures contribute to the development of next-generation optical sensors (optasensors) with enhanced sensitivity and signal transduction capabilities [14]. Additionally, electrochemical sensors with improved biocompatibility and electron transfer efficiency can be designed by harnessing biomineralization strategies [15]. Finally, the field has witnessed the emergence of smartphone-assisted biosensors [16] utilizing biomineral-based components, enabling user-friendly and field-deployable sensing solutions [17]. Collectively, these advancements underscore the transformative potential of biomineral-based biosensors in healthcare. Their application holds promise for earlier diagnoses and the development of personalized treatment strategies. Furthermore, real-time and accurate pollutant detection capabilities contribute to promoting environmental sustainability.

In this review, we aim to comprehensively illuminate the remarkable potential of biomineral-based biosensors. By delving into past advancements and diverse applications, we explore the utilization of established biominerals alongside emerging strategies employing bioinspired mineral structures. Furthermore, the review sheds light on the key principles underlying the construction of engineered mineral-based biomaterials. We highlight the unique physicochemical properties these materials possess, their intriguing ability for self-assembly into functional sensors, and the strategies employed for tailored functionalization. By exploring these aspects, we aim to provide a deeper understanding of the transformative potential of biomineral-based biosensors for revolutionizing healthcare, environmental monitoring, and diverse analytical applications.

## 2. Silica Biominerals and Their Biomimetic Analogues for Biosensing Applications

### 2.1. Biomineralized Silica Structures as Platforms for Biosensors

Biological silicification is a remarkable process found in various organisms, enabling the meticulous deposition and organization of silica into intricate structures under tight biological control [18]. This process is particularly prevalent in sponges, diatoms, and some plants. Silica deposition occurs through the precipitation of silicic acid (a soluble form of silica) within specialized compartments [4]. Siliceous sponges are marine multicellular organisms possessing skeletal frameworks consisting of silica-based, needle-like structures known as spicules [19]. These spicules provide structural support and defense mechanisms for the sponge [20]. Silica deposition in sponges is controlled by specialized cells called sclerocytes, which secrete organic matrices rich in silicateins and other proteins [21,22,23,24]. These proteins facilitate the nucleation and growth of silica particles, leading to the formation of spicules with diverse shapes and sizes. On the other hand, diatoms, a type of single-celled algae, are renowned for their intricate biosilica cell walls, known as frustules [25]. Notably, these frustules often possess hierarchical pore patterns, with diameters ranging from nano- to micrometers. Diatoms regulate the formation of their frustules within specialized compartments termed silica deposition vesicles (SDVs) through specialized organic matrices. These matrices include key players like polyamines, which are long-chain organic molecules rich in positively charged amine groups [26]. Polyamines work synergistically with other organic components, like silaffins [27] and silacidins [28], to control silica deposition and guide the morphogenesis of the frustule. Silaffins and silacidins, which are proteins rich in charged amino acids, play a crucial role in biosilicification [29,30,31,32]. Within SDVs, these proteins interact with silicic acid, catalyzing its polymerization and deposition [33]. This intricate interplay between organic and inorganic components ultimately orchestrates the formation of the frustule, a species-specific biosilica structure with exquisite geometric patterns that govern silica deposition and guide frustule morphogenesis [4].

Due to their intricate nanostructured morphology, high surface area, and species-specific architecture, diatom frustules show significant potential in biosensor applications [34]. These biosilica-based microstructures offer several advantages for biosensing, including their biocompatibility, chemical stability, and ease of functionalization [35]. Additionally, the porous nature of diatom biosilica allows for efficient immobilization of biomolecules such as enzymes, antibodies, and DNA probes, enhancing the sensitivity and specificity of biosensor devices [30,33,36]. Furthermore, the precisely controlled synthesis of diatom biosilica through biological processes enables the creation of tailored structures with desired properties, making them ideal candidates for the development of novel biosensing platforms with enhanced performance and versatility (Table 1).

**Figure 1:**
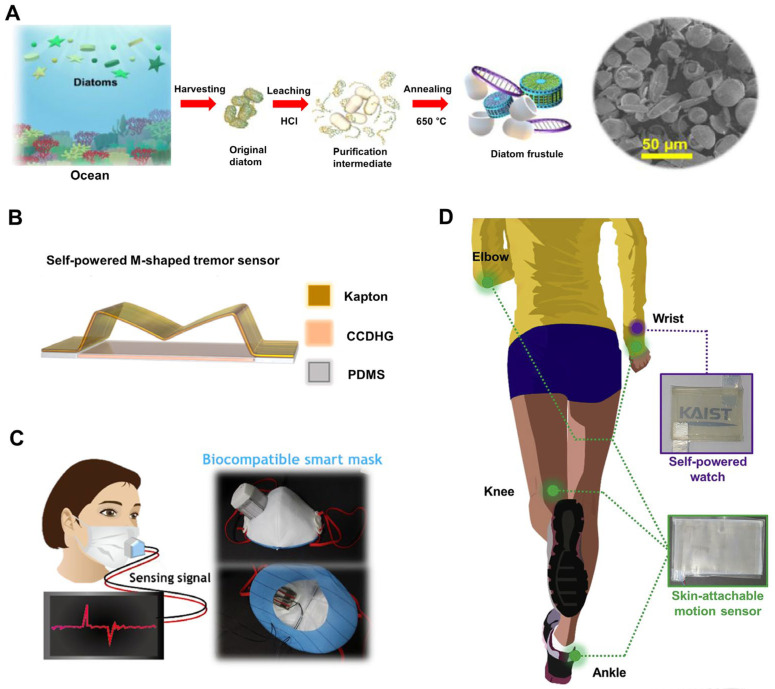
(**A**) Schematic illustrating the process for mass-producing diatom biosilica from cultivated diatom microalgae. Potential applications of diatom-based triboelectric nanogenerators (TENGs) in (**B**) a tremor sensor; (**C**) a self-powered, biocompatible, innovative mask design; and (**D**) a skin-attachable power generation device. (**A**,**C**): Reproduced with permission from [38], Copyright 2021 American Chemical Society. (**B**): Reproduced with permission from [37], Copyright 2021 Elsevier Ltd. (**D**): Reproduced with permission from [39], Copyright 2020 Elsevier Ltd.

The rising demand for real-time health monitoring has spurred significant advancements in wearable sensor technology, driven by the limitations of conventional sensors, such as external power dependencies and mechanical vulnerabilities. Interestingly, diatom biosilica presents a promising solution (Figure 1). One study introduced a highly stretchable and self-healing hydrogel conductor, CCDHG, synthesized from marine biomaterials including catechol, chitosan, and diatom biosilica [37]. This CCDHG enabled the creation of a stretchable triboelectric nanogenerator (TENG) for mechanical energy harvesting from human motion, boasting impressive output metrics such as an open-circuit voltage of 110 V and a short-circuit current of 3.8 μA. It was successfully deployed as a skin-attachable, self-powered tremor sensor for Parkinson’s disease (PD) patients (Figure 1B). Another study focused on enhancing cellulose nanofibril (CNF)-based TENGs by incorporating diatom biosilica (DB), resulting in remarkable output characteristics, including an output voltage of 388 V and a time-averaged power of 85.5 mW/m^2^ [38]. This DB-CNF TENG has found application in a self-powered smart mask for human breathing monitoring (Figure 1C), showcasing the diverse potential of diatom biosilica in wearable health technologies. Additionally, a study proposed the use of diatom biosilica and chitosan for developing skin-attachable TENGs (Figure 1D), yielding a significantly improved power density of 15.7 mW/m^2^ compared to pure chitosan TENG [39]. These studies underscore the potential of diatom biosilica in advancing TENGs and wearable biosensors, offering biofriendly and biocompatible solutions for energy harvesting and health monitoring.

Another intriguing application of diatom biosilica lies in its in vivo functionalization for biosensors targeting specific pathogens. A study explored this approach for improving the efficacy of biosensors targeting *Bacillus anthracis*, the causative agent of anthrax [40]. Prior attempts utilizing single-domain antibodies (sdAbs) against a surface-layer protein (EA1) faced limitations in detecting both intact spores and vegetative cells, hindering the biosensor’s utility for environmental monitoring of *B. anthracis*. To address this challenge, the researchers strategically modified the biosilica-targeting peptide Sil3T8. They retained the essential ER trafficking sequence at the N-terminus while relocating Sil3T8 to the C-terminus of the fusion protein. This strategic modification successfully enabled the detection of EA1 with both sdAbs, highlighting the critical role of Sil3T8 in achieving functional biosilica-localized biosensors. Furthermore, homology modeling suggested that the improved performance arose from the elimination of steric hindrances between the antigen-binding loops and the diatom biosilica. This finding underscores the significance of meticulous structural considerations in optimizing the functionality of biosilica-based biosensors, paving the way for advancements in environmental monitoring of *B. anthracis* and potentially other pathogens.

**Table 1:** Biominerals and their biomimetic analogues for biosensing applications.

Mineral Name	Sensor Type	Sensor Platform	Receptor	Target	Limit of Detection	Reference
Silica	Immunosensor	Diatom biosilica frustules	sdAb against *Bacillus anthracis* S Layer protein EA1	*Bacillus anthracis*	ND	[40]
	Photoluminescence immunosensor	Diatom biosilica functionalized with polydopamine and AuNPs	Goat anti-rabbit IgG	Rabbit IgG	8 × 10^−9^ mg mL^−1^	[41]
	Immunoassay (SERS)	Diatom biosilica with self-assembled plasmonic nanoparticles	Goat anti-mouse IgG	Mouse IgG	10 pg mL^−1^	[42]
	Immunoassay (fluorescence spectroscopy and imaging)	Diatom biosilica	Antibody against mouse IgG	Mouse IgG	140 fg mL^−1^	[43]
	Optical biosensor	Biomimetic silica nanoparticles	Intracellular enzyme system from *Raoultella planticola*	Nitrite	0.25 mg L^−1^	[44]
	Amperometric biosensor	Graphite rod electrode	Biosilica-entrapped GOx	Glucose	0.67 mM	[45]
	Amperometric biosensor	GCE	Biosilica-entrapped mSOx	Sarcosine	340 μM	[46]
	Amperometric biosensor	Gold nanoparticles	Biosilica-entrapped AChE	Acetylcholine	ND	[47]
Calcium carbonate	Electrochemical biosensor	Au-nanoparticle-coated chicken eggshell composite (Au/CaCO_3_ nanocomposite)	N/A	4-Nitrophenol	0.54 nM	[48]
	Electrochemiluminescence immunosensor	Au/CaCO_3_ nanocomposite (eggshell template)	Nanobody (conjugated to Au/CaCO_3_)	Ochratoxin A	5.7 pg mL^−1^	[49]
	Electrochemical aptasensor (sandwich-type)	Coccolith-modified screen-printed gold electrode	Cognate pair of aptamers	Vaspin (type 2 diabetes biomarker)	298 pM	[50]
Magnetosome	Whole-cell magnetic biosensor	Magnetotactic bacteria	Arsenic-inducible promoters	Arsenite	10 nM	[51]
	Magnetic ELISA	Magnetosome–nanobody complex	Anti-TBBPA nanobody	TBBPA	0.025 ng mL^−1^	[52]
	Magnetic ELISA	Magnetosome–nanobody complex	Anti-fipronil nanobody (requires oxidation for binding)	Fipronil (insecticide)	2.74 ng mL^−1^	[53]
	Magnetic immunoassay	BMPs modified with streptavidin	Biotinylated anti-3-PBA Nb2	3-PBA	0.03 ng mL^−1^	[54]
	ELISA/magneto-impedance spectroscopy	Magnetosome–antibody complex	Anti-*Salmonella* antibody	*Salmonella typhimurium*	10^1^ CFU mL^−1^	[55]
	ELISA/magneto-impedance spectroscopy	Magnetosome–antibody complex	Anti-listeriolysin antibody	*Listeria monocytogenes*	10^1^ CFU mL^−1^	[56]
	Magnetic concentration/qPCR detection	Biomimetic magnetic nanoparticles (BMNPs) synthesized with MamC protein	N/A (direct electrostatic interaction)	*Staphylococcus aureus*	10 CFU mL^−1^	[57]
	Nucleic acid detection	Magnetically driven bacterial microrobot	Cas12a protein	Decapod iridescent virus 1 (DIV1)	8 copies μL^−1^	[58]
	Nucleic acid detection	Magnetically driven bacterial microrobot	Cas12a protein	White spot syndrome virus (WSSV)	7 copies μL^−1^	[58]
Hydroxyapatite	Electrochemical aptasensor (sandwich-type)	GCE	Anti-PDGF-BB aptamer-modified HAP NPs	PDGF-BB	50 fg mL^−1^	[59]
	Electrochemical biosensor	GCE/HAp/rGO/AuNPs	Uricase enzyme	Uric acid	3.9 × 10^−6^ M	[60]
	Electrochemical (label-free)	Magnetic GCE	Fe_3_O_4_@HAP	miRNA let-7a	0.051 fM	[61]
	Electrochemical immunosensor	HAP-Au/SPCE	Receptor binding domain of spike protein	IgG antibodies against SARS-CoV-2	0.0561 pg mL^–1^	[62]
	Electrochemical aptasensor (sandwich-type)	GCE/graphene	MNP-TBA1/HAP-TBA2	Thrombin	0.03 fM	[63]

N/A, not applicable; ND, not determined; AuNPs, gold nanoparticles; qPCR, quantitative polymerase chain reaction; GCE, glassy carbon electrode; GOx, glucose oxidase; mSOx, monomeric sarcosine oxidase; IgG, immunoglobulin G; PDGF-BB, cancer biomarker platelet-derived growth factor-BB; HAP, hydroxyapatite; NPs, nanoparticles; BMPs, bacterial magnetic nanoparticles; TBBPA, tetrabromobisphenol A; NB2, bivalent nanobody; 3-PBA, 3-phenoxybenzoic acid; MNP, magnetic nanoparticles; TBA1, thrombin aptamer 1; TBA2, thrombin aptamer 2; sdAb, single-domain antibody; SPCE, screen-printed carbon electrode; rGO, reduced graphene oxide.

Diatom biosilica also holds promise for constructing highly sensitive immunoassay biosensors due to their unique photonic crystal properties [42]. These natural structures can significantly enhance the surface plasmon resonance of self-assembled silver nanoparticles (NPs) deposited on their surfaces, acting like light-amplifying lattices. This amplification dramatically increases the sensor’s sensitivity in SERS, a potent method for molecule detection based on unique light interactions. The researchers meticulously assembled the sensor in a series of steps. First, they attached cleaned diatom frustules (*Pinnularia* sp.) onto a glass slide. Next, they decorated the frustules with amino groups to facilitate strong electrostatic interactions with subsequently physisorbed silver NPs. This step was followed by the selective attachment of goat anti-mouse IgG antibodies to the silver NPs. Finally, they blocked non-specific binding sites using the BSA protein. The introduction of gold plasmonic nanoparticles labeled with both antibodies and the signal-enhancing molecule DTNB further amplified SERS signals. This designed sensor exhibited superior selectivity, binding only to the target molecule (mouse IgG) and achieving an impressive LOD of 10 pg/mL, surpassing conventional SERS sensors by a remarkable 100-fold. The study underscores the potential of hybrid plasmonic–biosilica nanostructures for advancing next-generation biosensors with ultrasensitive and highly specific detection capabilities. These advancements hold promise for significant applications in medical diagnostics and environmental monitoring.

**Figure 2:**
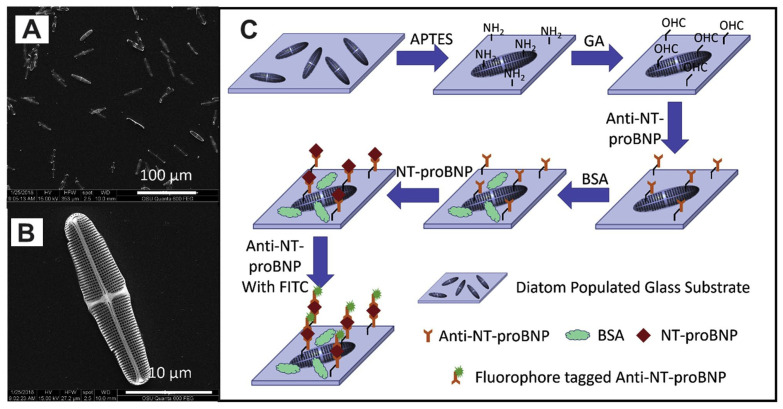
Scanning electron microscopy (SEM) images of (**A**) a densely packed diatom assembly on a glass slide and (**B**) a single diatom frustule. (**C**) Schematic representation of a diatom-based immunoassay for NT-proBNP detection. (**A**–**C**): Reproduced with permission from [64], Copyright 2019 Elsevier B.V.

Squire et al. explored the application of unaltered *Pinnularia* sp. diatoms in photonic crystals to enhance fluorescence for detecting mouse immunoglobulin G (IgG) protein in an immunoassay [43]. Following a standard antibody–antigen-labeled antibody protocol, they investigated how diatom biosilica photonic structures affected the fluorescence intensity of both rhodamine 6G (R6G) dye and R6G-labeled mouse IgG immunocomplexes attached to the diatom shells. By analyzing the fluorescence response at varying antigen concentrations, Squire et al. demonstrated a remarkable 100-fold improvement in the detection limit of fluorescence spectroscopy. This translated to a LOD of 14 fg/mL for mouse IgG, highlighting the significant fluorescence enhancement and high sensitivity achieved through this diatom-based immunoassay approach. Building upon this success, Squire et al. later employed the same diatoms in a fluorescence imaging immunoassay for detecting cardiovascular disease biomarkers (Figure 2) [64]. The focus shifted to detecting N-terminal pro-B-type natriuretic peptide (NT-proBNP), a critical biomarker for heart disease, showcasing the potential of these bio-inspired structures in disease diagnosis. The researchers employed functional groups on the diatom frustules to enable specific binding of anti-NT-proBNP antibodies while blocking non-specific interactions. The introduction of fluorescently tagged antibodies allowed for detection through the measurement of emitted fluorescence. This innovative approach using diatom frustules holds significant promise for the development of sensitive and specific diagnostic tools, potentially advancing early detection and improving patient outcomes across various diseases.

Inspired by naturally occurring diatom frustules, a novel biosensing platform was developed for highly sensitive immunodetection utilizing photoluminescence (PL) [41]. This platform leveraged diatom biosilica (DB), the primary constituent of frustules (as described earlier), which possesses a unique structure composed of porous hydrogenated amorphous silica with periodic micro- and nanoscale features. To enhance the platform’s functionality, the DBs were functionalized with gold nanoparticles (AuNPs) through a sophisticated in situ deposition process on a polydopamine (PDA) coating. This process significantly reduced the inherent blue PL intensity of DBs. Subsequently, the resulting DB-PDA-AuNPs were further functionalized with goat anti-rabbit immunoglobulin G (IgG), creating an antibody-decorated platform. The study demonstrated the key finding that specific binding with the target antigen, rabbit IgG, led to a significant increase in peak PL intensity compared to non-binding interactions. Notably, the enhanced PL intensity exhibited a linear correlation with the target antigen concentration, achieving a remarkable limit of detection (LOD) of 8 × 10^−6^ mg/mL. Further optimization led to an even lower LOD of 8 × 10^−9^ mg/mL, spanning an impressive eight orders of magnitude in sensitivity. This research underscores the potential of DBs as a powerful tool for label-free, PL-based immunoassays.

Building upon the presented research, diatom biosilica has emerged as a remarkably versatile and promising platform for advancing biosensor development. Its intricate nanostructure, high surface area, biocompatibility, and ease of functionalization make it a valuable tool for researchers. It enhances detection in immunoassays, facilitates label-free strategies, and even integrates into biocompatible wearables for health monitoring. Notably, its targeted pathogen detection capabilities showcase promise in environmental monitoring and beyond.

### 2.2. Biomimetic Silica for Biosensing Platforms

Beyond the utilization of natural diatom frustules, researchers are actively exploring ways to harness the power of biosilicification to create even more sophisticated biosensors. This involves mimicking natural biosilicification processes to fabricate hybrid biomimetic silica materials [65,66,67,68]. These engineered materials hold immense promise as they can potentially combine the well-established advantages of diatom biosilica, like high surface area and intricate structures [69], with additional functionalities specifically tailored for targeted biosensing applications. This bioinspired approach opens exciting avenues for the design of next-generation biosensors with enhanced sensitivity, selectivity, and biocompatibility (Table 1).

**Figure 3:**
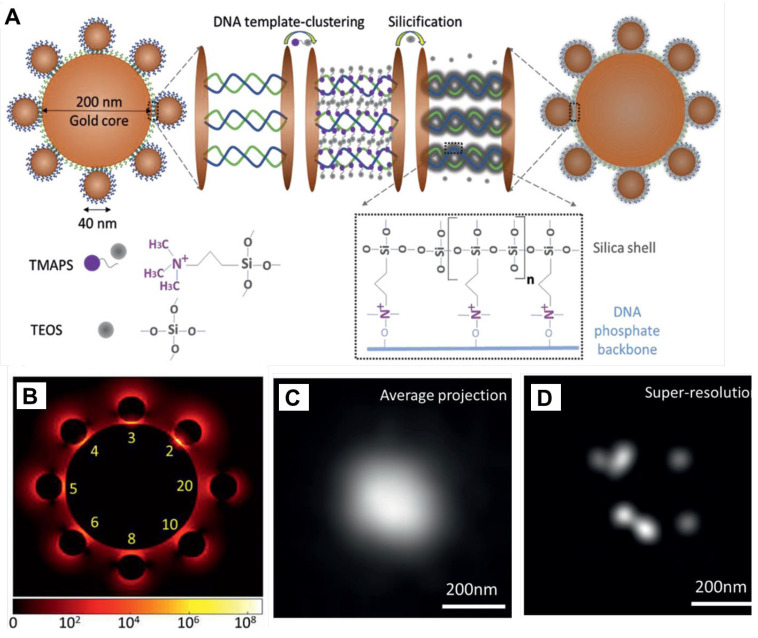
Design and fabrication of DNA-silicified templates for Raman optical beacons (DNA-STROBE). (**A**) Schematic of DNA-mediated silicification for core–satellite gold assemblies. (**B**) SERS enhancement mapping for DNA–silica hybrid gold assemblies with different core-satellite distances, indicated by yellow numbers in nanometers. (**C**) Diffraction-limited and (**D**) super-resolution imaging of plasmonic hotspots on DNA-STROBE. (**A**–**D**): Reproduced with permission from [14], Copyright 2021 Wiley-VCH GmbH.

Silica-based plasmonic nanocavities hold immense potential for optical sensing, but limitations exist in their structural stability and hotspot visualization. Liang et al. addressed these challenges by introducing DNA-silicified templates for Raman optical beacons (DNA-STROBE) (Figure 3) [14]. This method utilized DNA scaffolds to precisely organize plasmonic nanoparticles, significantly enhancing both structural stability and chemical inertness. Furthermore, DNA silicification enabled precise control over nanogaps, leading to substantial local electromagnetic field enhancement. This advancement allowed for single-molecule detection via SERS even at high background concentrations, overcoming typical limitations. Super-resolution SERS measurements further enabled noninvasive spatial profiling of plasmonic hotspots. DNA-STROBE’s programmability, reproducibility, and quantitative label-free readouts provided a versatile platform with broad applications in nanophotonics and biomedical research. This approach represents a significant leap forward, merging the strengths of DNA silicification and shell-isolated nanoparticle-enhanced Raman spectroscopy. DNA-STROBE has opened up exciting avenues for exploring both geometric and molecular properties within plasmonic nanocavities, paving the way for advancements across the biomedical field.

Another intriguing application has emerged from the utilization of silica biomineralization processes in the development of a novel amperometric biosensor design. In this research endeavor, covalently bonded acetylcholinesterase (AChE) was immobilized onto gold nanoparticles (Au NPs) to bolster stability and electron transfer [47]. To provide additional protection for the enzyme, a biomimetic silica layer was grown around the Au NP–enzyme complex, safeguarding it against denaturation and protease attacks. Validation of each construction step was conducted using ATR-FT-IR spectroscopy, while the biosensor’s remarkable long-term stability over four months was affirmed through amperometric evaluation. Notably, the biosilica nanocomposites encapsulating Au NPs-AChE conjugates not only facilitated efficient signal mediation but also markedly enhanced enzyme stability compared to conventional AChE biosensors, underscoring the critical role of biosilica in extending biosensor functionality and driving progress in bioanalytical applications.

A biomimetic silicification approach guided by silaffin peptides has emerged as a promising strategy for next-generation biosensor development, as evidenced by recent investigations. This technique leverages the unique self-assembly and silica-nucleating properties of silaffin peptides to engineer novel biofunctional materials.

Recently, Chen et al. investigated the potential of a monomeric sarcosine oxidase (mSOx) fused with the silica-forming peptide, silaffin R5, for biosensor development in resource-limited settings [46]. Although mSOx possesses inherently low activity, requiring high enzyme concentrations for amperometric biosensor signals within the clinically relevant range (<1 mM sarcosine), the researchers explored strategies to improve its dynamic range using an amperometric biosensor model. The research compared various mSOx constructs, including those tagged with either a 6x histidine (6H) tag or an R5 silaffin peptide, against the native enzyme. Glutaraldehyde-crosslinked proteins exhibited reduced activity, while R5-mediated biosilification on particles resulted in a self-immobilization matrix with improved enzyme activity. Using a silica precursor, tetramethoxysilane, a thick silica deposition layer was formed on a glassy carbon electrode, enabling mediated current with a dynamic range suitable for sarcosine detection (sarcosinemia). Furthermore, the mSOx-R5 fusion protein enabled biosilification with additional enzymes, achieving a mediated enzyme-linked current with a dynamic range suitable for creatinine detection. This study highlights the potential of the mSOx-R5 fusion protein for biosensor applications, particularly in resource-limited settings, for the clinical detection of sarcosine and creatinine. However, further optimization and real-world implementation studies are necessary.

A glucose biosensor with improved performance was developed by employing biomimetic silicification [45]. The researchers constructed a fusion protein by combining glucose oxidase (GOx), a critical enzyme for glucose detection, with the R5 silaffin peptide. This self-assembling fusion protein underwent biosilicification within a controlled environment, resulting in the encapsulation of GOx within a silica matrix. This biomimetic immobilization technique offered significant advantages compared to conventional methods, leading to improved stability and functionality of the biosensor. The resulting glucose biosensor exhibited a linear response for detecting glucose concentrations ranging from 0 to 1 mM, with a LOD of 0.67 mM. Additionally, it demonstrated exceptional stability and selectivity, even in the presence of interfering molecules commonly encountered in biological samples.

Building upon the development of biomimetic silicification for biosensor improvement, another study investigated the applicability of biomimetic silicification for gravimetric biosensors [70]. The researchers engineered a novel fusion protein by combining a silaffin peptide with green fluorescent protein (GFP). This protein conjugate was subsequently immobilized on a gold quartz crystal resonator. Upon exposure to a silicic acid solution, the specific interaction between the silaffin peptide and silica precursors facilitated the selective deposition of silica particles on the gold surface. This biomimetic approach resulted in a significant and quantifiable increase in the resonance frequency of the resonator, serving as a clear indicator of successful silicification. Notably, the absence of a frequency shift when employing bare GFP as a control further underscores the crucial role of the silaffin peptide in promoting silica formation.

**Figure 4:**
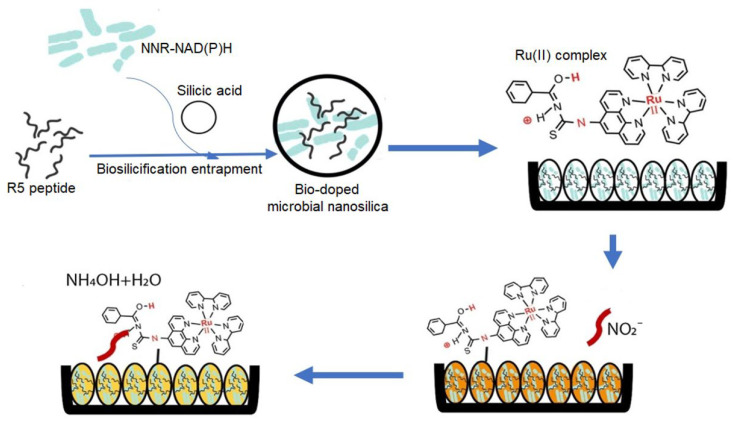
Schematic representation of a biomimetic optosensor for nitrite detection. Silaffin R5 peptide-guided silica encapsulation of NAD(P)H and nitrite reductase (NNR) enzyme. Ruthenium complex (pH label) on the silica surface. Nitrite reduction by NNR leads to color change (from orange to yellow) via reflectance. Reproduced with permission from [44], Copyright 2022 MDPI (CC BY 4.0).

A novel microbiologically derived optosensor was constructed for the selective and sensitive detection of nitrite ions (Figure 4) [44]. This optosensor leveraged biomimetic silica nanoparticles doped with R5, a silaffin peptide, to create a robust encapsulation matrix for *Raoultella planticola* cells and the essential cofactor NAD(P)H. The optosensor utilized a chromophoric pH indicator molecule immobilized on the biogenic nanosilica. Upon exposure to nitrite, the intracellular enzymatic machinery of the entrapped bacteria catalyzed its reduction, resulting in a localized pH shift that is detected by the indicator. This pH change was subsequently transduced into a measurable optical signal via reflectance. The microbial optosensor exhibited a linear response to nitrite concentrations ranging from 1 to 100 mg L^−1^, with a low LOD of 0.25 mg L^−1^ and a rapid response time of 4 min. It also demonstrated excellent stability, high precision, and a minimal response to common interferences encountered in processed meat samples.

Bioinspired silica nanotubes, synthesized through a templated approach inspired by natural biomineralization processes, were investigated as a novel sensing platform for explosives [71]. Self-assembled, amyloid-like peptide nanostructures served as templates, guiding the controlled growth of silica nanotubes via amine group interactions with the silica precursor. The resulting highly porous bioinspired silica nanotubes were subsequently functionalized with a fluorescent dye, enabling the development of a sensitive nitro-explosive vapor detection platform. The fabricated thin films exhibited rapid, selective, and efficient detection through a fluorescence quenching mechanism upon exposure to targeted explosives. This work demonstrates the remarkable potential of bioinspired silica nanotubes, templated by designed peptides, for practical applications in explosives detection. Their unique structural properties and selective fluorescence quenching response offer a promising avenue for developing advanced explosive sensing devices.

Collectively, these studies demonstrate the remarkable potential of biomimetic silicification as a versatile platform for developing high-performance biosensors. This approach offers distinct advantages in terms of enhanced sensitivity, stability, and selectivity for various biosensing applications, with demonstrated effectiveness in both enzymatic and non-enzymatic sensing strategies. However, as highlighted in the research, further optimization and real-world implementation studies are necessary to fully realize the potential of this promising technology.

## 3. Calcium Carbonate Biominerals and Their Biomimetic Analogues for Biosensing Applications

### 3.1. Biomineralized Calcium Carbonate Structures as Platforms for Biosensors

Biological calcification processes encompass various organisms and structures with unique characteristics and functions [72]. These processes lead to mineral deposition under physiological conditions, forming essential structures such as bones, teeth, and shell materials like mollusks and eggshells, as well as coccoliths, corals, and pearls [73]. One notable example is the formation of eggshells, which occurs in birds, reptiles, and some fish species. Eggshells are primarily composed of calcium carbonate (CaCO_3_) in the form of calcite crystals, arranged in a matrix of proteins and polysaccharides [74,75]. This structure provides protection for developing embryos while allowing for gas exchange. Similarly, coccoliths are intricate calcium carbonate plates produced by single-celled marine algae known as coccolithophores [76]. These microscopic organisms play a crucial role in marine ecosystems and carbon cycling. Coccoliths are arranged in elaborate patterns around the surface of the algal cell, forming a protective armor-like structure that helps regulate buoyancy and provides defense against predators and environmental stresses [76]. Mollusks, one of the most diverse groups of animals in terms of species richness, also utilize calcium carbonate for shell formation [77]. Mollusk shells vary widely in size, shape, and composition, reflecting the diverse habitats and lifestyles of these organisms. For example, gastropods like snails and slugs typically have coiled shells, while bivalves such as clams and oysters have hinged shells composed of two halves or valves [78]. Their intricate compositions and arrangements serve various roles in protection, support, and survival for the organisms that produce them.

Importantly, due to their unique properties, these biomineralized CaCO_3_ structures hold potential for applications in biosensors. For example, the porous nature of eggshells allows for gas exchange, suggesting potential applications in gas-sensitive biosensors [79,80]. With their intricate patterns and armor-like structure, coccoliths could inspire the design of robust and protective sensor coatings [81]. Mollusk shells, with their diverse shapes, sizes, and compositions, offer a wealth of materials for biomimetic sensor development, potentially enabling the creation of sensors with tailored properties for specific applications [82]. Overall, exploring the utilization of these natural structures in biosensor technology holds promise for advancing sensor design and functionality (Table 1).

Recent studies have demonstrated the transformation of readily available eggshells (CaCO_3_) into gold nanoparticle composites (Au/CaCO_3_), unlocking new possibilities for environmental remediation and food safety. This sustainable approach offers a cost-effective method for waste management and the creation of powerful sensor-based tools [48,49]. The first study explored Au/CaCO_3_ for environmental remediation, targeting the pollutant 4-nitrophenol (4-NP) [48]. The developed composites functioned as a dual-function sensor and catalyst. This translated to highly accurate detection of 4-NP at low concentrations through the sensor function while simultaneously promoting its breakdown through the catalyst function. This work tackled waste management and offered a sustainable solution for environmental cleanup. The second study investigated Au/CaCO_3_ for food safety. A novel biosensor based on electrochemiluminescence (ECL) technology utilized these composites [49]. The large surface area of eggshell-derived Au/CaCO_3_ allowed for effective immobilization of detection molecules, leading to a highly accurate and reliable sensor for ochratoxin A in food. This research highlighted the potential of eggshells for advancing food safety measures in a cost-effective manner. These studies demonstrated the remarkable potential of transforming eggshells into functional nanomaterials with significant applications in environmental and health domains, paving the way for a more sustainable future with improved sensor technology.

**Figure 5:**
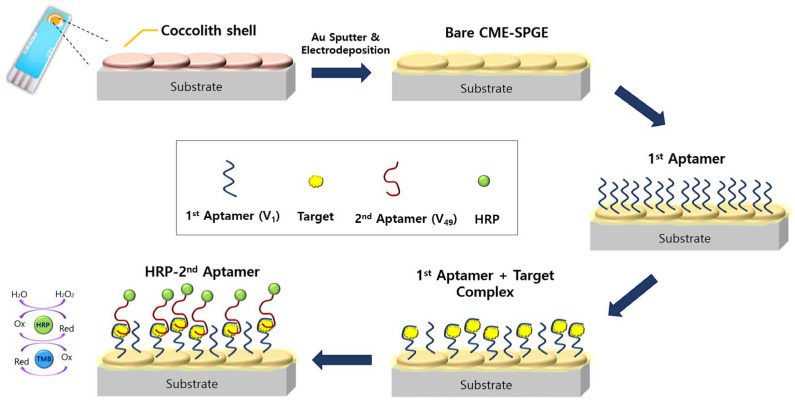
Fabrication of a coccolith-modified electrochemical aptasensor. The process of creating a coccolith-modified electrodeposited (CME) onto a screen-printed gold electrode (SPGE) is shown. The CME-SPGE serves as the platform for a subsequent cognate pair aptamer-based electrochemical aptasensor. Reproduced with permission from [50], Copyright 2018 Elsevier B.V.

Intricate biogenic calcite microparticles, known as coccoliths, offer a unique platform for biosensor development due to their highly textured morphology. Kim et al. explored a pioneering approach by utilizing coccoliths derived from the biomineralizing alga *Emiliania huxleyi* to fabricate a sandwich-type electrochemical biosensor for vaspin, a biomarker associated with type 2 diabetes mellitus (Figure 5) [50]. The process involved modifying a screen-printed gold electrode (SPGE) with coccoliths through drop-coating, followed by the deposition of a gold (Au) thin film via sputtering and subsequent Au electrodeposition to enhance conductivity and stability. The resulting electrochemical aptasensor on the coccolith-modified electrodeposited SPGE (CME-SPGE), integrating a cognate aptamer pair, demonstrated exceptional selectivity and sensitivity for vaspin detection, with a remarkable LOD of 298 pM and an extended linear range. This innovative strategy established the coccolith-modified SPGE with a cognate aptamer pair as a promising platform for designing high-performance electrochemical biosensors.

The exploration of bivalve mollusks as biosensors presents another avenue for leveraging CaCO_3_-based structures in environmental monitoring [83]. Bivalves exhibit a distinctive behavioral response characterized by increased valve gape in the presence of waterborne pollutants, providing a quantifiable indicator of pollution levels. Ahmmed et al. integrated inertial measurement units (IMUs) into their biosensing system to precisely measure the valve-gape angle, correlating directly with the extent of environmental contamination and enabling real-time monitoring of water quality [83]. By simultaneously monitoring multiple bivalves housed within water-insulated sensor units and wirelessly transmitting data to a central base station for analysis, the system ensured comprehensive monitoring without disrupting the natural behavior of the organisms. Benchtop testing confirmed the system’s precision in angle measurements, while in vivo experiments validated the consistent translation of valve-gape behavior into quantifiable data. This approach presents a promising avenue for utilizing bivalves as environmental sentinels, offering a potentially cost-effective and real-time method for water quality assessment.

Building upon the presented research, the exploration of CaCO_3_ as a platform for biosensors represents a significant advancement in sensor technology. Through the utilization of naturally occurring biomineralized structures like eggshells, coccoliths, and mollusk shells, researchers have showcased the versatility and potential of CaCO_3_-based biosensing.

### 3.2. Biomimetic Calcium Carbonate for Biosensing Platforms

**Figure 6:**
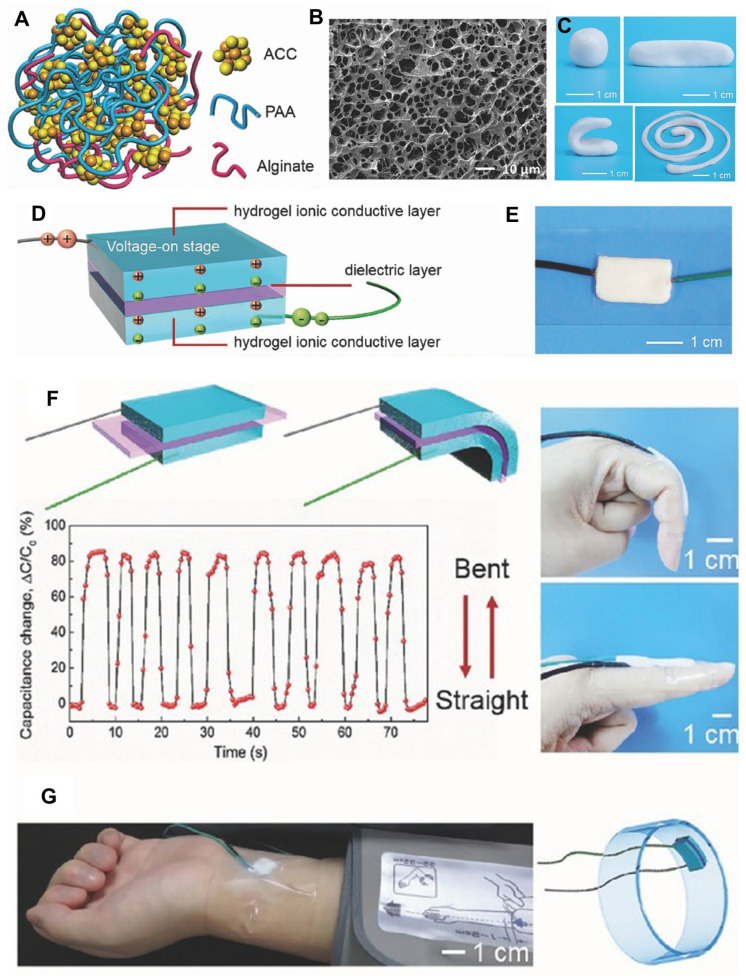
Bioinspired calcium carbonate-based ionic skin. (**A**) Schematic of the biomimetic hydrogel (ACC/PAA/alginate). (**B**) SEM image of the porous hydrogel structure. (**C**) Shape-adaptable hydrogel for versatile sensor designs. (**D**) Working principle: charge accumulation at interfaces upon voltage application for pressure detection. (**E**) Photograph of a sensor fabricated with biocompatible hydrogel. (**F**) Finger motion sensor: hydrogel sensor responds to finger bending with real-time capacitance changes. (**G**) Blood pressure sensor design and real-time signal variation reflecting changes in arm pressure. (**A**–**G**): Reproduced with permission from [84], Copyright 2017 WILEY-VCH Verlag GmbH & Co. KGaA, Weinheim.

Recent advancements in biomimetic approaches have revolutionized biosensor development, effectively transcending the limitations of naturally occurring CaCO_3_ biominerals. These strategies exploit the unique physicochemical properties of CaCO_3_, meticulously replicating the exquisite control observed in natural calcification processes. This approach empowers researchers to engineer novel biosensing platforms with enhanced functionalities and superior performance capabilities. For instance, a highly sensitive, autonomously self-healable ionic skin was developed using a bioinspired supramolecular mineral hydrogel (Figure 6) [84]. This hydrogel incorporated nanoscale amorphous calcium carbonate (ACC) particles physically crosslinked with alginate chains and polyacrylic acid (PAA). This innovative design facilitated the fabrication of a novel class of mechanically adaptable ionic skin sensors. Due to its unique viscoelastic properties, the resulting capacitive sensor exhibited superior compliance, self-healing capabilities, and the ability to detect subtle pressure variations, including gentle finger touches, human motion, and even minute water droplets. Notably, the sensor boasted a high-pressure sensitivity reaching 1 kPa. This biomimetic approach, employing a mineral hydrogel-based ionic skin, holds immense promise for the development of next-generation intelligent skin-like devices with superior softness, stretchability, and mechanical adaptability, paving the way for advancements in artificial intelligence, human–machine interaction, personal healthcare, and wearable technology applications.

Moreover, researchers have developed a novel biomimetic hydrogel fortified with stabilized ACC nanoparticles achieved through dual-ion doping (PO_4_^3−^ and Mg^2+^) [85]. This approach extended the applications of flexible wearable strain sensors and artificial skin. This mineral hydrogel exhibited exceptional properties, including remarkable extensibility (>1150% strain), significantly enhanced fracture toughness, and desirable linear strain sensitivity. Furthermore, the hydrogel demonstrated excellent biocompatibility and flame retardancy, making it a promising candidate for wearable device applications. The strain sensor derived from this hydrogel showcased high sensitivity and accuracy in detecting a range of human activities. This research established a compelling strategy for creating biomimetic hydrogels with superior mechanical properties and sensing performance, paving the way for their use in advanced wearable strain sensors.

Further highlighting the versatility of ACC in biomimetic sensor design, Li et al. introduced a novel method for crafting a multifunctional epidermal sensor that addressed key challenges in reliable healability, robust mechanical properties, environmental degradability, and efficient sensing capabilities [86]. This innovative sensor relied on a highly stretchable, self-healing, degradable, and biocompatible nanocomposite hydrogel achieved through the meticulous conformal coating of a MXene (Ti3C2Tx) network within hydrogel polymer networks. Importantly, the incorporation of ACC enhanced the sensor’s mechanical properties and contributed to its biocompatibility. The multifunctional epidermal sensor exhibited remarkable sensitivity in detecting human motions with a rapid response time (20 ms) and served as an electronic skin for wirelessly monitoring electrophysiological signals, such as electromyogram and electrocardiogram signals. Notably, the sensor’s degradability in a phosphate-buffered saline solution ensures minimal environmental impact. The MXene-PAA-ACC hydrogel, with its augmented mechanical properties and self-healing capabilities, holds tremendous promise for applications in human–machine interactions, personalized health diagnostics, and smart robot prostheses, showcasing its potential as a flexible and wearable multifunctional epidermal sensor.

Amorphous calcium carbonate (ACC) has garnered significant attention in biomimetic materials research due to its unique properties and wide-ranging applications. Researchers recently introduced an innovative method to stabilize ACC using carboxylated cellulose nanofibrils (CNFs), drawing inspiration from the natural stabilizing effect observed in crustacean cuticles [87]. By harnessing the synergistic impact of abundant carboxyl groups and the rigid nanofibril geometry of CNFs, this approach achieved superior stabilization efficiency, surpassing its synthetic and biopolymeric counterparts. The resulting CNF/ACC hybrid facilitated the fabrication of transparent composite films with remarkable mechanical properties, boasting a strength of 286 MPa and toughness up to 28.5 MJ/m^3^, surpassing previously reported materials. This study underscores the sustainability and biological basis of CNF/ACC composites and highlights their versatile functionalities, positioning them as promising materials for diverse applications in biomedicine, packaging, and wearable devices.

Building upon the limitations of naturally occurring CaCO_3_ biominerals, researchers have engineered novel biosensing platforms with enhanced functionalities and capabilities. This breakthrough was achieved by leveraging the unique properties of CaCO_3_ through biomimetic approaches. These approaches meticulously mimic the exquisite control exhibited by natural calcification processes. Consequently, the reliance on natural CaCO_3_ biominerals has been surpassed, paving the way for the development of next-generation biosensors with superior performance.

## 4. Magnetosome Biominerals and Their Biomimetic Analogues for Biosensing Applications

### 4.1. Biomineralized Magnetosome Structures as Platforms for Biosensors

Magnetotactic bacteria, a unique group of motile, Gram-negative microorganisms, possess the remarkable ability to synthesize specialized organelles known as magnetosomes [88,89]. These fascinating biomineralized compartments house magnetic nanocrystals, showcasing a sophisticated interplay between biological control and material properties. The size and morphology of magnetosomes exhibit significant interspecies variability, typically ranging from 35 to 120 nm [90,91]. The precise molecular mechanisms governing magnetosome biomineralization remain under active investigation. However, researchers have identified specific proteins, such as Mms5, Mms6, Mms7, and Mms13, that localize to the magnetosome membrane in certain magnetotactic bacterial strains [7,92,93]. Notably, Mms6, a small amphiphilic protein, plays a pivotal role in magnetosome membrane localization and regulates its formation and growth rate [94]. This protein exhibits a well-defined structural organization, featuring an N-terminal domain forming a hydrophobic core and a C-terminal domain harboring a specific iron-binding motif (DEEVE) [95,96]. This motif is believed to be essential for initiating the nucleation and subsequent growth of magnetite crystals within the magnetosome. Additionally, the highly conserved MamC (Mms13) protein, prevalent across magnetotactic alpha-proteobacteria with a size of approximately 12.4 kDa, suggests a crucial yet enigmatic role in magnetosome biomineralization [97,98].

Exploring the realm of magnetosome-based biosensors, a novel magnetic biosensor for ultrasensitive arsenic detection in water contamination has been developed by genetically modifying magnetotactic bacteria (Table 1) [51]. Employing a transcriptional fusion technique, researchers integrated the bacteria’s arsenic-inducible promoters with the bacterial luciferase (luxCDABE) operon. This ingenious engineering enabled the bacteria to luminesce upon arsenic exposure, providing a readily quantifiable signal. The sensor exhibits exceptional sensitivity, achieving a LOD of 10 nM, surpassing World Health Organization (WHO) guidelines for safe drinking water. This was accomplished through a two-step process: initial detection by the genetically modified bacteria (0.5 micromolar), followed by magnetic concentration for signal amplification. Additionally, the sensor delivered rapid results (30 min for fresh cells, 90 min for freeze-dried cells) and boasted remarkable bacterial robustness, even enduring freeze-drying for storage and transport. The inherent magnetic properties of the bacteria further enhanced the technology by enabling effortless separation from the water sample via magnetic concentration and streamlining analysis. Overall, this magnetic biosensor presented significant progress for arsenic detection, offering high sensitivity, rapid detection times, bacterial robustness, and simplified analysis through magnetic separation.

**Figure 7:**
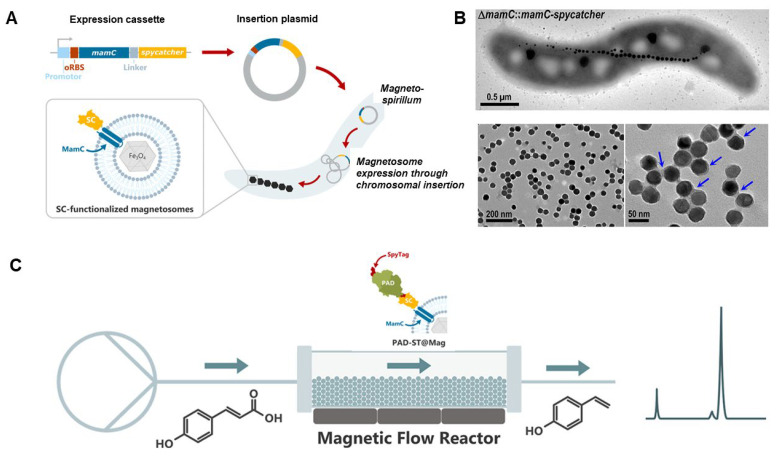
(**A**) Bioengineered SpyCatcher-functionalized magnetosomes from Magnetospirillum gryphiswaldense. (**B**) Transmission electron microscopy (TEM) images of isolated magnetosomes obtained from mutant *M. gryphiswaldense* cells. The organic shell, corresponding to the magnetosome membrane, is indicated by blue arrows. (**C**) Schematic depiction of the biocatalytic application. The engineered magnetosomes are coupled with SpyTag-fused dimeric phenolic acid decarboxylase (PAD-ST) to facilitate the conversion of p-coumaric acid to p-hydroxystyrene within a magnetic microreactor in flow. (**A**–**C**): Reproduced with permission from [99], Copyright 2022 American Chemical Society (CC BY 4.0).

Further expanding the applications of magnetosome-based biosensors, SpyCatcher magnetosomes served as a transformative platform for the development of biocatalytic nanoparticles (Figure 7) [99]. This innovative approach significantly expanded the available genetic toolbox for engineering magnetic nanoparticles with tailored functionalities. The core technology lay in the SpyTag–SpyCatcher system, a versatile strategy that enabled the rapid and specific attachment of diverse proteins to the magnetosomes. This flexibility unlocked a broad spectrum of applications in biocatalysis, particularly benefiting enzymes that function optimally within a membrane environment. Beyond the SpyCatcher system, the inherent properties of magnetosomes, including high crystallinity, stable magnetic moments, and narrow size distribution, rendered them ideally suited for biocatalyst immobilization via genetic engineering. The researchers developed a modular connector system where abundant magnetosome membrane anchors were genetically fused with SpyCatcher coupling groups. This facilitated efficient covalent coupling with complementary SpyTag-functionalized proteins. The resulting SpyCatcher magnetosomes demonstrated superior performance compared to commercially available particles. They exhibited exceptional stability and superior substrate conversion rates over a 60 h period, highlighting the effectiveness of this approach.

In the domain of magnetosome-based biosensors, magnetosome–nanobody complexes demonstrate high binding affinity for environmental pollutants, paving the way for novel enzyme-linked immunosorbent assays (ELISAs) [52]. Researchers utilized this binding ability to develop an ELISA approach for the detection of tetrabromobisphenol A (TBBPA), an environmental pollutant. The magnetosome–Nb-based ELISA demonstrated high sensitivity and specificity, with a LOD of 0.025 ng/mL. The magnetosome–Nb complex could be reused multiple times without compromising assay performance. This biosensor offered advantages such as cost-effectiveness, environmental friendliness, and reusability, making it a promising tool for environmental monitoring, food safety, and medical diagnostics.

Magnetosome-based biosensors are experiencing significant advancements, leading to the development of highly sensitive immunoassays for environmental monitoring. These assays target both pollutants and their corresponding biomarkers of exposure, highlighting the versatility and potential of this innovative technology. Wu et al. explored the utilization of magnetosomes in immunoassays for insecticide fipronil detection in water samples [53]. The researchers employed genetically engineered magnetotactic bacteria to produce Nb–magnetosomes, which were subsequently conjugated with camel-derived nanobodies. The CFFF bacterial strain yielded Nb–magnetosomes with demonstrably superior fipronil binding affinity. Interestingly, oxidized Nb–magnetosomes exhibited enhanced recognition of unconjugated fipronil while displaying reduced binding to fipronil-HRP. This finding enabled the development of a tailored ELISA for fipronil detection in water, achieving recoveries between 78% and 101%. This cost-effective and environmentally benign approach positions Nb–magnetosomes as a promising platform for nanobody-based fipronil immunoassays, with broader applicability to other Nb-based assays. Additionally, a novel immunomagnetic bead-based immunoassay for detecting 3-phenoxybenzoic acid (3-PBA), a biomarker for pyrethroid exposure, utilized biotinylated bivalent nanobodies (Nb2) immobilized on streptavidin (SA)–modified bacterial magnetic nanoparticles (BMPs) [54]. The BMP–SA–biotin–Nb2 complexes demonstrated exceptional stability under harsh conditions, including extreme pH, methanol, and high ionic strength. The assay exhibited high sensitivity and accuracy for 3-PBA detection, with an IC50 value of 1.11 ng/mL. Notably, the assay successfully analyzed spiked sheep and cow urine samples, achieving quantitative recoveries ranging from 82.5% to 113.1%. These results were highly consistent with those obtained using liquid chromatography–tandem mass spectrometry (LC–MS/MS). The BMP–SA–biotin–Nb2-based immunoassay offered several advantages, including its high sensitivity, specificity, tolerance for harsh conditions, operational simplicity and speed, and cost-effectiveness. This approach allowed for direct 3-PBA analysis in urine samples, eliminating the need for complex pretreatment steps such as alkaline neutralization following acidic deconjugation. Overall, the BMP–SA–biotin–Nb2-based immunoassay emerges as a promising tool for rapid, reliable monitoring of 3-PBA in urine samples, with potential applications in various scenarios requiring 3-PBA detection.

In a comprehensive examination of magnetosome applications in biosensing, researchers have showcased the versatility and efficiency of magnetosome-based biosensors in detecting various microorganisms in food and water (Table 1). Sannigrahi et al. pioneered a magnetosome–anti-*Salmonella* antibody complex biosensor utilizing *Magnetospirillum* sp. RJS1-derived magnetosomes [55]. This cost-effective and sensitive method for detecting *Salmonella typhimurium*’s lipopolysaccharide held promise for practical *Salmonella* detection. The same research group applied a similar magnetosome–antibody complex strategy for rapid *Escherichia coli* detection in contaminated food samples, likely employing an ELISA or analogous immunoassay technique [100]. Sannigrahi et al. focused on *Listeria monocytogenes* detection, presenting a magnetosome-based biosensor with high sensitivity (LOD of 10^1^ CFU/mL) in water and milk samples [56]. Utilizing magnetosomes coupled with antibodies targeting listeriolysin—a virulence factor produced by Listeria—the biosensor likely immobilized the magnetosome–antibody complex on an electrode surface. This configuration facilitated label-free detection, eliminating the need for additional markers, by measuring changes in electrical impedance upon target capture. Additionally, a novel electrochemical immunosensor for detecting staphylococcal enterotoxin B in milk incorporated bio-magnetosomes alongside a polyaniline nano-gold composite and an ionic liquid [13]. This intricate design demonstrated enhanced sensitivity, stability, and specificity compared to traditional methods. The specific detection protocol relied on antibody–antigen interaction between staphylococcal enterotoxin B and the biosensor surface, leading to measurable changes in electrical properties. A recent study focusing on the detection of white spot syndrome virus (WSSV) in seafood samples further enriched this perspective by introducing an innovative magnetosome-based impedimetric biosensor [101]. This approach, utilizing magnetosomes functionalized with VP28-specific antibodies, exhibited notable sensitivity and specificity in WSSV detection, contributing significantly to the advancing body of research in this field. Collectively, these studies underscore the promise of magnetosome-based biosensors for diverse microorganism detection, addressing crucial concerns related to food safety and water quality.

### 4.2. Biomimetic Magnetosomes for Biosensing Platforms

Building upon the exciting potential of biocatalytic magnetosomes, researchers are exploring even more ingenious approaches. One such approach involved MamC-targeted biomimetic magnetic synthesis, offering a novel route for biosensor applications [102,103]. The development of biomimetic magnetic nanoparticles (BMNPs) through MamC-targeted synthesis presented a novel approach to biosensor applications. Unlike conventional magnetic nanoparticles (MNPs), BMNPs enabled direct electrostatic interaction with microorganisms due to their unique surface characteristics, eliminating the need for post-production coating steps [57]. This feature enhanced the binding efficiency of BMNPs, facilitating the concentration and detection of bacteria in fluid samples. Moreover, BMNPs exhibited remarkable versatility, effectively binding to a wide range of bacteria, including both Gram-positive and Gram-negative types. Specific detection of target microorganisms such as *Staphylococcus aureus* at concentrations as low as 10 CFU/mL was achieved using quantitative polymerase chain reaction (qPCR), demonstrating the system’s potential for various diagnostic applications [57]. Additionally, researchers have explored the use of BMNPs in nanoassemblies for detecting contaminants in aqueous samples [104]. These nanoassemblies, utilizing either inorganic or biomimetic magnetic nanoparticles, served as substrates to immobilize enzymes such as acetylcholinesterase (AChE) and β-lactamase (BL). Optimization of the nanoassembly involved exploring enzyme immobilization through electrostatic interaction or covalent bonds, with covalent nanoassemblies exhibiting superior sensitivity. The enzymatic stability was ensured by setting parameters such as temperature, ionic strength, and pH, while the enzyme load on the nanoparticles allowed preserved activity to reach 50–60% of the free enzyme’s specific activity. The covalent nanoassemblies exhibited remarkable sensitivity, detecting trace concentrations of pollutants as low as 1.43 nM chlorpyrifos and 0.28 nM penicillin G while also enabling quantification of higher concentrations. Furthermore, enzyme immobilization provided enhanced stability to AChE and allowed for BL reuse up to 12 cycles. This research highlights the efficacy of BMNPs as biosensors for detecting pollutants in water, offering a promising avenue for environmental monitoring and remediation efforts.

**Figure 8:**
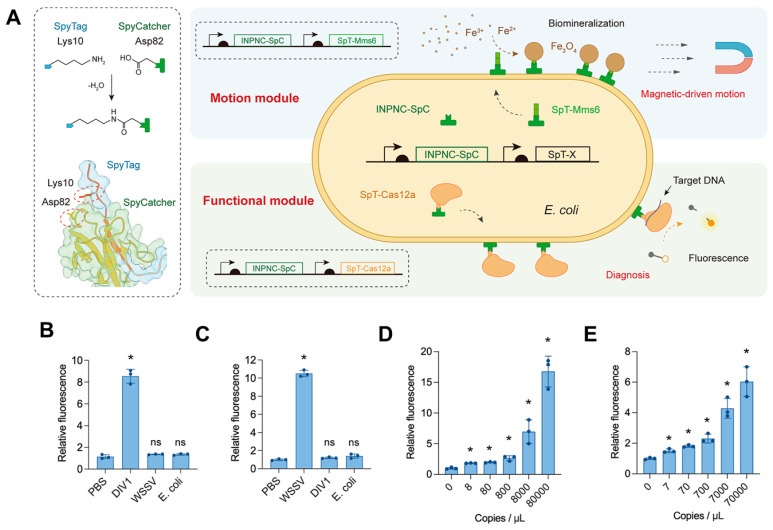
Bioengineered bacterial microrobots for multifunctionality. (**A**) Modular design using SpyCatcher/SpyTag for surface display of functional proteins (Cas12a for DNA detection, Mms6 for biomineralization) on bacterial microrobots. Specificity (**B**,**C**) and sensitivity (**D**,**E**) of detection of DIV1 and WSSV nucleic acid sequences, respectively. * *p* < 0.05, ns not significant. Reproduced with permission from [58], Copyright 2023 American Chemical Society.

Another innovative approach involved targeting the Mms6 protein for biomimetic magnetic synthesis directly on the cell surface, opening new avenues for biosensor applications. An extraordinary breakthrough in this field was the creation of magnetically driven diagnostic bacterial microrobots, which function as microscopic biosensors for rapid and accurate pathogen detection in aquaculture [58]. These microrobots, synthesized using advanced synthetic biology techniques, represented a convergence of mobility and targeted functionality. A key advancement lay in the incorporation of surface-displayed Mms6 protein, enabling the microrobots to form a magnetic shell responsive to external magnetic fields (Figure 8). This feature ensured precise magnetic steering with a remarkably high magnetization intensity of 18.65 emu g^−1^, enhancing control during navigation within samples. Additionally, the diagnostic capability was facilitated by another engineered surface protein, Cas12a, enabling the microrobots to detect specific DNA sequences from aquatic pathogens with exceptional sensitivity and accuracy. The achieved detection limits (8 copies μL^−1^) for decapod iridescent virus 1 (DIV1) and 7 copies μL^−1^ for white spot syndrome virus (WSSV) significantly exceeded requirements for reliable field testing and exhibited high concordance with traditional diagnostic methods when applied to real shrimp tissue samples. This development approach leveraged a versatile Spy system within synthetic biology, enabling efficient display of functional proteins on the bacterial surface and creating modular building blocks for these microrobots. Beyond their diagnostic capabilities, these magnetic microrobots offered additional advantages, including responsiveness to controlled magnetic motion for precise sample navigation and reusability, making them a cost-effective and sustainable solution for aquaculture pathogen detection.

## 5. Hydroxyapatite Biominerals and Their Biomimetic Analogues for Biosensing Applications

Hydroxyapatite (HAP), a calcium phosphate bioceramic with a nanoscale structure, offers exceptional biocompatibility and even promotes bone formation [105]. Its bioactivity, chemical similarity to bone, and osteoconductivity make it ideal for bone repair applications [106]. Interestingly, HAP possesses piezoelectricity and pyroelectricity, properties associated with certain biominerals, which may contribute to bone healing by responding to mechanical stress [107]. Inspired by this intricate biofabrication process in nature, scientists are actively mimicking biomineralization to engineer HAP with tailored properties for diverse applications beyond bone repair. These applications include electrocatalysis, photocatalysis, and drug delivery due to its adsorption and ion exchange capabilities [108]. It is also a popular scaffold material in tissue engineering [108]. While pure HAP suffers from low mechanical strength, incorporating reinforcing materials creates high-performance composites. In recent years, HAP has seen a surge in its use for biosensors, optical devices, bioimaging, and implant coatings [109]. The ability of HAP to create a biocompatible microenvironment in biosensors makes it ideal for immobilizing biomolecules on electrochemical sensors. Furthermore, its superior biocompatibility, adsorption capacity, stability, and sensitivity make it a superior choice for biosensor fabrication. The utilization of nanoscale HAP, with its augmented surface area, amplifies its potential in biosensor applications (Table 1) [109].

Recent studies have demonstrated the effectiveness of HAP-based biosensors in various applications, showcasing their adaptability and superior performance. For instance, hydroxyapatite-based electrochemical aptasensors exhibited enhanced sensitivity through innovative dual-signal amplification strategies [59]. The innovative approach involved utilizing hydroxyapatite nanoparticles (HAP-NPs) as a substrate, enabling the sequential deposition of anti-platelet-derived growth factor-BB (PDGF-BB) antibody, PDGF-BB analyte, and anti-PDGF-BB aptamer onto a glassy carbon electrode, creating a sandwich structure. Both HAP-NPs and the aptamer incorporated phosphate groups, allowing for the generation of a redox-active molybdophosphate precipitate on the GCE surface. The resulting amperometric signal exhibited linearity within the 0.1 pg/mL to 10 ng/mL PDGF-BB concentration range, achieving an impressive detection limit of 50 fg/mL. The adaptability and versatility of this strategy extended its potential to various electrochemical biosensing applications for biomarkers and related species. The study underscored the importance of addressing challenges related to HAP nanoparticle surface functionalization and stability in aqueous solutions for future advancements in this promising field.

Another study explored a ternary nanocomposite composed of HAP, gold nanoparticles, and reduced graphene oxide to enhance uricase biosensor performance [60]. Electrochemical evaluation of the modified glassy carbon electrode revealed improved electrocatalytic activity for uric acid detection. The UAO (uricase)/HAP-rGO/AuNPs sensing system exhibited a linear relationship between peak current intensity and a broad uric acid concentration range (1.95 × 10^−5^ to 6.0 × 10^−3^ M). It achieved a low LOD of 3.9 × 10^−6^ M and a high analytical sensitivity of 13.86 mA/M. The biosensor accurately determined uric acid concentrations in human urine samples. This uricase biosensor, characterized by its biocompatibility and strong electrochemical performance, presents a promising and practical approach for rapid and accurate clinical analysis of uric acid levels.

**Figure 9:**
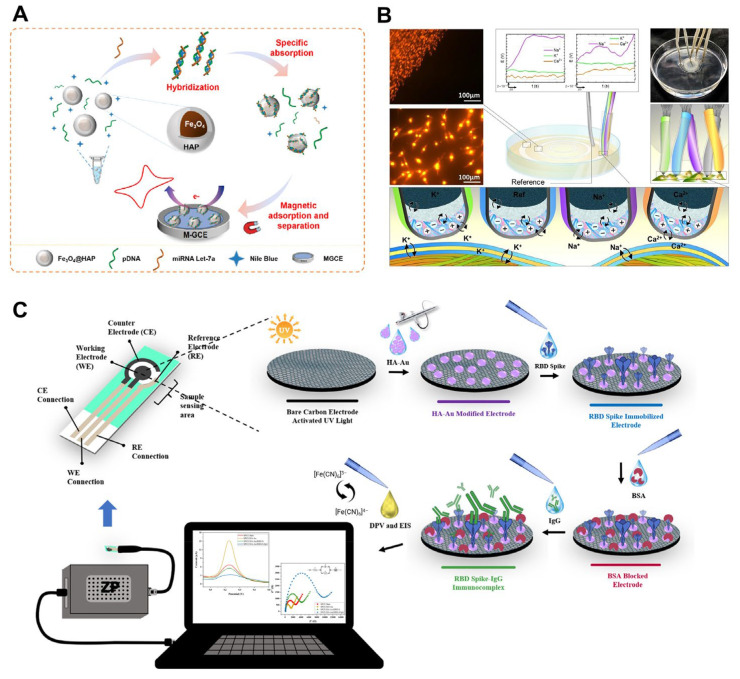
Versatile biomimetic hydroxyapatite for biosensing applications. (**A**) Magnetic HAP-based electrochemical biosensor for label-free detection of miRNA Let-7a. (**B**) Piezoresponsive HAP patterns on a biomimetic platform facilitate sensitive recording of extracellular ion currents for studying cell-to-cell communication. (**C**) HAP-Au-modified electrode for selective detection of SARS-CoV-2 antibodies via an immunosensing approach. (**A**): Reproduced with permission from [61], Copyright 2023, American Chemical Society. (**B**): Reproduced with permission from [110], Copyright 2024 Elsevier Ltd. (CC BY-NC-ND 4.0). (**C**): Reproduced with permission from [62], Copyright 2024 American Chemical Society.

Additionally, label-free detection of microRNA let-7a was achieved using hydroxyapatite-based electrochemical biosensors, demonstrating high sensitivity and specificity (Figure 9A) [61]. The innovative design exploited the inherent selectivity of HAP nanoparticles for double-stranded nucleic acids, eliminating the need for intricate probe immobilization or labeling procedures. The sensing mechanism involved introducing a target-specific probe DNA strand, which, upon encountering its complementary microRNA let-7a, underwent hybridization to form a double helix. This double-stranded complex subsequently underwent selective capture by the HAP nanoparticles. The final step involved the introduction of Nile blue, a dye molecule exhibiting strong binding affinity towards the paired bases within the DNA duplex. This interaction generated a robust electrical signal that was readily measured by the sensor. The exceptional sensitivity (LOD of 0.051 fM) and specificity achieved by this approach could be attributed to the synergistic effects of target–probe hybridization, the dye’s interaction with the double-stranded structure, and the selective adsorption of HAP towards double-stranded DNA. This label-free and immobilization-free strategy presents a promising avenue for facile and accurate microRNA analysis with potential applications in clinical diagnostics.

Innovative approaches involving hydroxyapatite patterns integrated with fiber-based biosensors have provided insights into cell-to-cell communication and tissue microenvironments (Figure 9B) [110]. This biocompatible system utilized self-assembled HAP patterns to replicate tissue microenvironments, enabling the study of spatially controlled waves of calcium, sodium, and potassium ions within cellular communities. The research demonstrated the ability of these HAP patterns to initiate calcium signaling pathways, with the application of norepinephrine leading to the propagation of electrical signals along the HAP structure. Fiber-based biosensors positioned above the patterns facilitated the simultaneous detection of various ion currents in the extracellular space. This work marked the first application of HAP patterns integrated with biosensors for studying cell-to-cell communication, offering a promising platform for future lab-on-a-chip devices and research into the fundamental mechanisms of cellular communication, particularly during regenerative processes.

Recently, hydroxyapatite–gold nanocomposite-modified immunosensors exhibited high sensitivity in detecting antibodies against SARS-CoV-2, the causative agent of COVID-19 [62]. The sensor utilized a meticulously modified screen-printed carbon electrode (SPCE) functionalized with a pre-optimized and characterized hydroxyapatite–gold nanocomposite (HAP-Au) (Figure 9C). This HAP-Au composite facilitated the direct spray-coating of the spike protein’s receptor binding domain (RBD), enabling efficient antibody immobilization. The SPCE/HAP-Au immunosensor exhibited a significantly enhanced current response compared to the unmodified electrode. Researchers rigorously evaluated the specific interaction between the immobilized RBD and immunoglobulin G (IgG) antibodies using advanced electrochemical techniques, including differential pulse voltammetry and electrochemical impedance spectroscopy. This approach yielded an impressive LOD of 0.0561 pg mL^−1^ for IgG antibodies, demonstrating the sensor’s high sensitivity. Furthermore, the SPCE/HAP-Au immunosensor boasted excellent selectivity and stability, retaining its efficacy for up to 7 weeks under controlled storage conditions. The sensor’s validity was rigorously confirmed by analyzing real human serum samples, with results demonstrating excellent concordance with those obtained via the established chemiluminescent microparticle immunoassay (CMIA) method. Overall, the SPCE/HAP-Au/RBD-S immunosensor exhibited exceptional characteristics of specificity, selectivity, and stability. These features position it as a promising candidate for the serological diagnosis of COVID-19. This innovative technology holds significant potential for future applications in evaluating the effectiveness of SARS-CoV-2 vaccination strategies.

Recent studies have also explored the integration of hydroxyapatite into homogeneous aptasensors for thrombin detection, resulting in ultraprecise and highly sensitive detection platforms. The initial investigation showcased an ultraprecise sandwich-type electrochemical aptasensor that harnessed the potential of HAP for heightened performance. This sensor integrated thrombin aptamer-functionalized magnetic nanoparticles (MNP-TBA1) and hydroxyapatite nanoparticles (HAP-TBA2) as dual recognition elements, with HAP playing a pivotal role in signal amplification [63]. The ensuing HAP-assisted sandwich structure enabled specific thrombin capture, while the incorporation of graphene significantly improved electron transfer efficiency, resulting in an impressive LOD of 0.03 fM. Subsequently, the second study introduced a hydroxyapatite-based dual-channel homogeneous aptasensor for the accurate quantification of proteins, particularly focusing on thrombin [111]. This sensor seamlessly merged colorimetric and electrochemical detection methods into a single platform, utilizing a G-quadruplex sequence for visual colorimetric detection and forming a HAP-supported sandwich structure for electrochemical current generation. This innovative approach achieved a low LOD of 0.40 fM and a wide linear range (0.1 fM to 1 nM), demonstrating robust performance in human serum analysis and highlighting its potential for swift and reliable biomolecule detection. Overall, these studies collectively emphasized the adaptability and promising prospects of homogeneous aptasensors, particularly those incorporating hydroxyapatite, opening avenues for the development of highly sensitive and innovative detection methodologies with significant applications in clinical diagnostics and biomarker analysis.

Researchers have leveraged the biocompatibility and stability of HAP, particularly when doped with gadolinium (Gd^3+^), to design a novel, high-performance MRI contrast agent (Apt-TDNs-GdHAP) for improving tumor cell imaging [112]. This innovative probe incorporated tetrahedral DNA nanostructures (TDNs) conjugated with a high-affinity aptamer (AS1411) that specifically targeted nucleolin, a protein overexpressed on tumor cell membranes. The TDNs facilitated the synthesis of highly water-dispersible nano-HAP and enhanced tumor cell recognition. As a result, Apt-TDNs-GdHAP probes exhibited a significant improvement in their ability to enhance signal intensity (r1 relaxivity) in MRI scans compared to traditional GdHAP probes, leading to superior T1-weighted images. Furthermore, the incorporation of TDNs bolstered the probe’s stability and its ability to specifically target tumors. This multifaceted design, combining the strengths of TDNs, aptamers, and HAP nanoparticles, holds promise as a powerful tool for MRI-based tumor cell imaging. This technology holds significant promise for revolutionizing cancer diagnosis, treatment monitoring, and drug delivery strategies.

## 6. Bioinspired Minerals and Materials as Platforms for Biosensors

Bioinspired mineral-based biosensors, particularly those employing non-natural biomineralization processes, are rapidly emerging as a transformative frontier in biomimicry and materials science. This burgeoning field leverages the exquisite control offered by synthetically engineered minerals to create novel biosensing platforms with exceptional capabilities. By drawing inspiration from, but not strictly replicating, nature’s intricate mineralization mechanisms, these biosensors offer significant enhancements in sensitivity, selectivity, and overall functionality. This development holds significant promise for advancements in various fields, such as diagnostics, environmental monitoring, and biomedical research.

Recent advancements in bioinspired mineral-based biosensors have highlighted their remarkable versatility and potential for various applications (Table 2). In one study, Nandini et al. demonstrated a novel synthesis method for one-dimensional gold nanostructures (AuNs) using β-diphenylalanine (β-FF) peptides as sacrificial templates [113]. These AuNs were then utilized in the development of highly sensitive cholesterol biosensors, showcasing their potential in biomedical diagnostics. Another noteworthy study introduced a bioinspired approach for synthesizing fluorescent gold nanoclusters (AuNCs), employing co-assembly molecules such as peptides and mercaptoundecanoic acid [114]. These AuNCs were integrated with graphene oxide, resulting in a sensitive “turn-on” fluorescent probe. This innovative biosensor exhibited particularly high sensitivity, particularly in detecting MMP-9, an enzyme biomarker associated with cancer. The potential of these nanoclusters extends to swift and cost-effective cancer diagnosis, presenting promising advancements in biomedical sensing technologies. Furthermore, a peptide-mediated strategy was employed to synthesize fluorescent copper nanoclusters (Cu NCs) utilizing the artificial peptide CLEDNN as a template [115]. These Cu NCs demonstrated high fluorescence quantum yield, excellent stability, and temperature-dependent fluorescent properties suitable for diverse applications, including cellular imaging and temperature sensing. This research underscores the versatility and broad utility of bioinspired mineral-based biosensors, offering insights into their potential for various biomedical and diagnostic applications beyond cancer detection.

Furthering the exploration of bioinspired mineral-based biosensors, an innovative colorimetric immunosensing method was developed for detecting carcinoembryonic antigen (CEA), a vital cancer biomarker [116]. This method combined the enzyme-induced biomineralization of cupric subcarbonate with a sensitive copper chromogenic reaction. Operating on a gold nanoparticle (Au NP)/urease nanoprobe modified with a signal antibody, the immunosensing mechanism orchestrated a sandwich immunoreaction on a magnetic bead (MB)-based platform. This precise capture of the target CEA and the Au NP/urease nanoprobe onto the MB surface initiated the enzyme reaction, generating ions that trigger biomineralization. Subsequently, Cu^2+^ release catalyzed a chromogenic reaction, resulting in a distinctive red complex. This approach enabled ultrasensitive detection of CEA, with a wide linear range spanning five orders of magnitude and a low LOD of 0.45 pg/mL. With its cost-effectiveness, rapid operation, and adaptability to detect other biomarkers, this method holds promise for early cancer diagnosis and monitoring in diverse clinical settings.

**Table 2:** Bioinspired minerals and materials as platforms for biosensors.

Mineral/Material	Sensor Type	Sensor Platform	Receptor	Target	Limit of Detection	Reference
Gold (peptide-templated	Electrochemical	Gr/GO-SH/AuNs	Cholesterol oxidase	Cholesterol	0.2 nM	[113]
Gold (peptide-templated	Fluorescence	Peptide–gold nanocluster/graphene nanocomplex	Peptide (specific to the MMP-9 cleavage site)	MMP-9	0.15 nM	[114]
Cobalt phosphate crystals	Enzymatic	Biomineralized OPH-fused bacteria	OPH enzyme	Paraoxon	0.08 μM	[117]
Fe-MOF	Optical (colorimetric)	Biomimetic mineralized HRP@Fe-MOF	Aptamer (specific to Aβ oligomer)	Aβ oligomer (associated with Alzheimer’s disease)	0.03 pM	[118]
Mineralized MOF	Electrochemical (competitive immunoassay)	Nanobody conjugated with biomimetic mineralized MOF	Nanobody (specific to Aflatoxin B1)	Aflatoxin B1	20.0 fg mL^−1^	[119]
Cu_3_(PO_4_)_2_	Electrochemical aptasensor	Cu_3_(PO_4_)_2_ nanoflowers/GO	Aptamer (specific to the carcinoembryonic antigen)	Carcinoembryonic antigen	2.4 fg mL^−1^	[120]
Ca_3_(PO_4_)_2_	Colorimetric (potentially)	Paper-based with PAL@Ca_3_(PO_4_)_2_ hybrid nanoflowers	PAL	Phenylalanine	60 μM	[121]
Cu_3_(PO_4_)_2_	Colorimetric (smartphone-assisted)	Laccase–mineral hybrid microflowers	Laccase enzyme	Epinephrine	0.6 μM	[122]

Gr, graphite; GO-SH, thiol-functionalized graphene oxide; AuNs, Au nanostructures; OPH, organophosphorus hydrolase; HRP, horseradish peroxidase; MMP-9, matrix metalloproteinase-9; MOF, metal–organic framework; Fe, iron; PAL, phenylalanine ammonia lyase; Cu_3_(PO_4_)_2_, copper phosphate.

**Figure 10:**
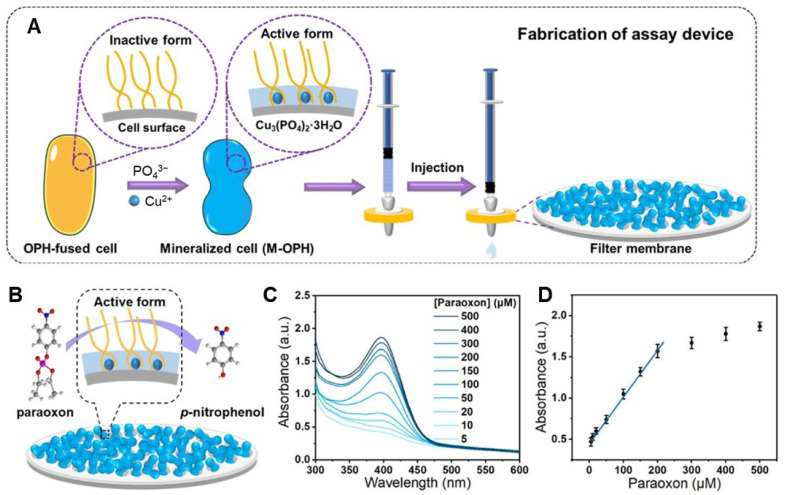
Biomineralized multifunctional device for detection of paraoxon. Schematic illustration of (**A**) the fabrication process and (**B**) the paraoxon assay on the resulting M-OPH-loaded membrane. (**C**) Absorbance spectra of filtrates obtained from incubating the M-OPH-loaded membrane with different paraoxon concentrations (5–500 μM). (**D**) Calibration curve for paraoxon detection using the M-OPH-loaded membrane. (**A**–**D**): Reproduced with permission from [123], Copyright 2021 Elsevier B.V.

The biomineralization of cobalt phosphate crystals onto the bacterial cell surface was explored to enhance the biosensing performance of organophosphorus hydrolase (OPH) [117]. This unique strategy created a bioinorganic hybrid catalyst with a distinct spindle-shaped morphology. The key to this innovation lay in the allosteric effect triggered by the cobalt phosphate shell. This shell essentially activated the previously inactive OPH entombed within, leading to a remarkable threefold increase in enzyme activity and improved stability compared to the free enzyme. This method resulted in a significantly more sensitive biosensing assay for paraoxon detection. Furthermore, researchers developed a user-friendly and portable detection device (Figure 10) [123]. This device is achieved through the straightforward sedimentation of the mineralized OPH-fused cells (M-OPH) on a simple syringe filter. The developed device achieved rapid analysis (50 s detection time), user-friendly operation requiring minimal training, excellent selectivity for paraoxon detection, practical sensitivity for real-world applications, and a visual readout for easy result interpretation. It successfully detected organophosphorus pesticides in various real samples, like watermelon, spinach, and cabbage juice. The bioinorganic approach holds promise for the development of cost-effective, eco-friendly biocatalysts impacting catalysis, biosensing, and real-time pollutant detection.

Innovative methods have emerged to improve enzyme stability and activity in biosensing applications. In a recent study, attention was drawn to enzyme encapsulation as a solution to the limitations of horseradish peroxidase (HRP) [118]. Through the utilization of iron-based metal–organic frameworks (Fe-MOFs), a protective shell was crafted around HRP molecules, closely emulating natural biomineralization processes [118]. This biomimetic encapsulation significantly enhanced the stability, sensitivity, and selectivity of HRP, allowing for notable enzymatic activity even at physiological temperatures. This approach demonstrated potential for the development of novel optical biosensors with sensitive spectral responses. Additionally, the integration of a nanobody (Nb) with a biomimetic mineralized MOF introduced a new strategy for creating immunoprobes [119]. This innovative design encapsulated succinylated HRP (sHRP) within mineralized MOF, amplifying sensitivity in immunoassays by addressing Nb’s limited signal reporter capacity. The developed electrochemical immunosensor demonstrated excellent performance in detecting aflatoxin B1 (AFB1), featuring wide linear ranges, high sensitivity, specificity, stability, and reproducibility. These findings not only broaden the applicability of Nbs but also introduce versatile concepts with potential applications to a wider range of biorecognition elements.

**Figure 11:**
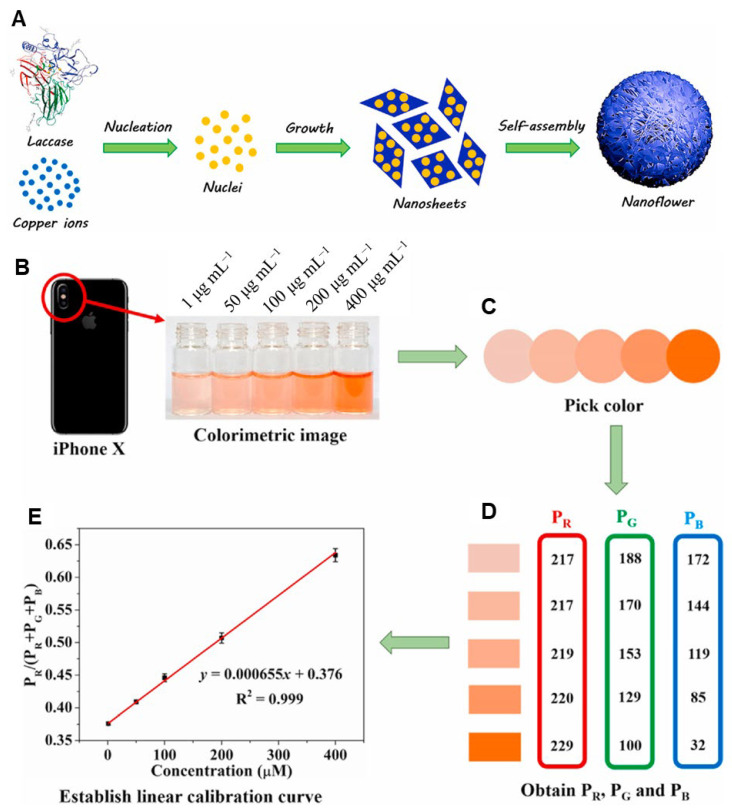
Laccase–mineral hybrid microflowers for smartphone-based colorimetric detection. (**A**) Schematic illustration of the process for creating laccase–mineral hybrid microflowers (La-HMFs). (**B**–**D**) La-HMF-based smartphone-assisted colorimetric detection: (**B**) colorimetric image captured by a smartphone camera; (**C**) color selection using a color picker application; (**D**) extraction of red (P_R_), green (P_G_), and blue (P_B_) pixel values from the chosen color; (**E**) establishment of a linear calibration curve for quantitative analysis. (**A**–**E**): Reproduced with permission from [122], Copyright 2020 Elsevier B.V.

Nanoflowers, intricately structured at a microscopic scale through biomineralization, have emerged as versatile platforms with diverse applications in biosensing and beyond. Among recent studies, one introduced a signal-quenching aptasensor for carcinoembryonic antigen (CEA) detection utilizing aptamer–copper phosphate (Cu_3_(PO_4_)_2_) hybrid nanoflowers with graphene oxide composites [120]. Despite signal suppression, the aptasensor demonstrated remarkable sensitivity for CEA detection. Additionally, disposable electrodes modified with biocompatible hybrid NFs synthesized from amino acids exhibited significant sensitivity and selectivity in detecting calf thymus double-stranded DNA (dsDNA) and the anticancer drug daunorubicin, contributing to a deeper understanding of anticancer drug–DNA interactions [124]. Furthermore, the development of flower-like, porous antimicrobial agents through a mineralization process incorporating organic polydopamine and inorganic copper phosphate components showed efficacy against Gram-negative *E. coli*, holding promise for clinical and industrial applications [125]. Moreover, a recent study addressed the need for simplified phenylketonuria (PKU) detection methods [121]. The research team developed a novel paper-based biosensor utilizing phenylalanine ammonia lyase (PAL) hybrid nanoflowers. This sensor demonstrated enhanced enzyme stability and rapid detection of phenylalanine in urine samples, offering a promising alternative to conventional PKU diagnostic techniques. These advancements showcased the versatility of nanoflowers in biosensing. Their applications ranged from disease marker detection and drug–DNA interaction analysis to the development of eco-friendly antimicrobial agents and accessible diagnostic tools. Furthermore, a novel smartphone-assisted portable biosensor was developed utilizing laccase–mineral hybrid microflowers (La-HMFs) for real-time and accurate quantification of epinephrine (EP) (Figure 11) [122]. La-HMFs were synthesized via biomineralization using the mineral copper phosphate, facilitating the immobilization of laccase molecules. The smartphone-assisted colorimetric assay provided a convenient and sensitive method for EP detection. This method featured a wide linear detection range (1–400 μM) for EP, impressive anti-interference capabilities, and a low LOD of 0.6 μM. This innovative biosensor offers a user-friendly and field-deployable approach for real-time epinephrine analysis.

The remarkable advancements in bioinspired mineral-based biosensors showcased in these studies underscore the transformative potential of non-natural biomineralization within biosensing applications. By harnessing synthetic mineralization processes, these biosensors have demonstrated remarkable advancements in sensitivity and functionality across diverse fields, including diagnostics, environmental monitoring, and biomedical research. The innovative strategies presented in these studies signal a paradigm shift towards more effective and adaptable biosensing platforms with the capacity to significantly enhance healthcare outcomes and promote environmental sustainability. Looking ahead, ongoing exploration and innovation in this area are imperative to unlocking further breakthroughs and paving the way for even more impactful contributions to healthcare and environmental protection.

## 7. Conclusions and Outlook

Biomineral-based biosensors represent a rapidly evolving field with significant potential to transform various scientific disciplines. By harnessing the unique properties of biominerals and mimicking nature’s exquisite biofabrication processes, researchers have engineered highly sensitive, selective, and functional biosensors. This review has comprehensively explored the diverse applications of biomineral-based biosensors in healthcare, environmental monitoring, and analytical fields. We have highlighted the utilization of established biominerals alongside the exciting possibilities offered by bioinspired mineral structures.

Looking towards the future, several key areas offer significant opportunities for further development. These advancements hold immense promise for real-world applications in various fields. One particularly promising direction lies in deeply harnessing the genes and proteins involved in biomineralization. Deciphering the intricate genetic and enzymatic pathways employed by organisms to construct complex biominerals can unlock a new level of biomimicry. This deeper understanding will guide the design of novel bioinspired structures with precisely tailored properties for biosensing applications, leading to the development of highly sensitive and specific sensors for early disease detection, environmental pollutant monitoring, and food safety analysis.

Moreover, integrating biomineral-based biosensors with microfluidics and nanotechnology presents another exciting avenue for advancement. This convergence has the potential to create miniaturized, high-throughput, and multiplexed sensing platforms, enabling the analysis of smaller sample volumes, faster processing times, and the simultaneous detection of multiple targets.

Enhancing biocompatibility and biorecognition remains a crucial area of exploration for biomineral-based biosensors. Continued research on surface functionalization strategies is essential to improve their biocompatibility and minimize tissue damage or rejection. However, these strategies can introduce unintended consequences. For instance, certain functional groups might trigger immune responses or hinder the sensor’s ability to interact with the target analyte [126]. Despite these challenges, surface functionalization offers a powerful tool for achieving high biorecognition [127]. By mimicking natural binding interactions, biomimetic approaches can enhance a sensor’s selectivity for specific biomarkers. However, optimizing these interactions for complex biological environments, which can introduce interferences that affect sensor performance, remains an active area of research.

Furthermore, the incorporation of machine learning and artificial intelligence algorithms offers a transformative leap in data analysis capabilities [128,129]. These algorithms can unlock new levels of data analysis, improve signal processing, and facilitate real-time monitoring, leading to faster and more accurate results. Finally, the development of user-friendly and portable biomineral-based sensors presents a paradigm shift for point-of-care diagnostics and environmental sensing. These user-friendly sensors could enable on-site and real-time detection in resource-limited settings, proving particularly beneficial for early disease detection and environmental monitoring efforts.

By addressing these key areas, biomineral-based biosensors have the potential to become a cornerstone technology for personalized medicine, environmental monitoring, and a wide range of analytical applications. The transformative potential of this technology promises to usher in a new era of sensitive, selective, and user-friendly biosensing.
